# Caveolin-3: A Causative Process of Chicken Muscular Dystrophy

**DOI:** 10.3390/biom10091206

**Published:** 2020-08-20

**Authors:** Tateki Kikuchi

**Affiliations:** Department of Animal Models for Human Disease, National Institute of Neuroscience, NCNP, Kodaira-shi, Tokyo 187-8502, Japan; pokkidog@jcom.zaq.ne.jp

**Keywords:** Caveolin-3, chicken muscular dystrophy, WWP1, β-dystroglycan, stretching

## Abstract

The etiology of chicken muscular dystrophy is the synthesis of aberrant WW domain containing E3 ubiquitin-protein ligase 1 (WWP1) protein made by a missense mutation of *WWP1* gene. The β-dystroglycan that confers stability to sarcolemma was identified as a substrate of WWP protein, which induces the next molecular collapse. The aberrant WWP1 increases the ubiquitin ligase-mediated ubiquitination following severe degradation of sarcolemmal and cytoplasmic β-dystroglycan, and an erased β-dystroglycan in dystrophic αW fibers will lead to molecular imperfection of the dystrophin-glycoprotein complex (DGC). The DGC is a core protein of costamere that is an essential part of force transduction and protects the muscle fibers from contraction-induced damage. Caveolin-3 (Cav-3) and dystrophin bind competitively to the same site of β-dystroglycan, and excessive Cav-3 on sarcolemma will block the interaction of dystrophin with β-dystroglycan, which is another reason for the disruption of the DGC. It is known that fast-twitch glycolytic fibers are more sensitive and vulnerable to contraction-induced small tears than slow-twitch oxidative fibers under a variety of diseased conditions. Accordingly, the fast glycolytic αW fibers must be easy with rapid damage of sarcolemma corruption seen in chicken muscular dystrophy, but the slow oxidative fibers are able to escape from these damages.

## 1. Introduction

In 1954, an animal model with inherited muscular dystrophy was found in a commercial flock of New Hampshire chickens. The poultry farmers brought affected birds to the department of poultry science (later changed to avian science) at the University of California at Davis. Drs. Asmundson and Julian, in the school of veterinary medicine, examined them clinically and pathologically. The early results were reported by Asmundson and Julian [1].

**Figure 1:**
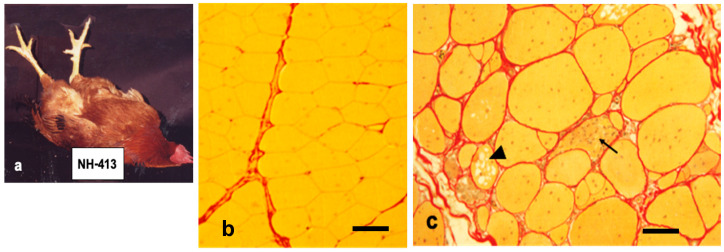
Chickens with muscular dystrophy (line 413) cannot right themselves from the spine position when they placed on their back while normal birds stand up instantly from this position (**a**). The pectoralis muscles from normal (**b**) and dystrophic (**c**) chickens at seven months are stained with Sirius Red. Normal pectoral muscle fibers (line 412) have polygonal contour and yellow cytoplasm outlined basal lamina by bright red line. They are wrapped by reddish connective tissue. Dystrophic pectoralis muscles are earliest and most severely affected, which are characterized by a marked variation in size with a proliferation of intracellular nuclei, necrotic phagocytosis (arrow), multivesicular fibers (arrow head) and fibrosis with lipid droplets. Note that dystrophic fibers lead to develop thicker endomysium layer compared to age matched wild-type ones. Bars in (b) and (c) indicate 80 and 100 μm, respectively.

Fertilized eggs from this source were used to establish a line homozygous for the abnormality. The most consistent clinical symptom was caused by the disfunction of the fast-twitch muscles with a predominance of fast-twitch αW and fast-twitch αR fibers [2], which correspond to the Type IIB and Type IIA fibers in mammalian muscles, respectively [3]. Pectoralis muscle (*M. pectoralis superficialis)* from breast part, PLD muscle (*M. posterior latissimus dorsi*) from the dorsal part and biceps muscle (*M. biceps brachii)* and patagialis muscle (*M. tensor patagiii longus*) from the fore arm and the patagium of the upper arm, respectively, are examples of chicken fast-twitch muscles which revealed severe pathological changes after hatching (Figure 1b,c). Among fast-twitch muscles, pectoralis muscle was the earliest and most severely affected soon after 2–3 weeks ex ovo. Chickens flap their wings using these muscles for flying up and right themselves instantly from the spine position when placed on their back [4]. Birds that could rise five times are assigned a score of 6 and considered normal. As the disease progresses, they fail to rise due to the inability to move breast and wing muscles. This movement performance is designated as flip test or exhaustion test to evaluate the disease progression and pharmaceutical effect (Figure 1a).

On the other hand, the mixed-fiber type muscles include, in addition to the fast-twitch fibers, the slow-twitch βR fibers or few slow-tonic α’ and β’ fibers. They localize mostly in the leg, neck and dorsal positions, and they are capable of retaining relatively normal state for months after hatching. The twitch fibers of chicken have a single motor end plate, whereas slow-twitch βR and slow-tonic fibers are multiply innervated [5,6]. The complexus muscle (*M. complexus*) from the neck is a mixed type, which contains, among the majority of fast-twitch fibers, a few slow-twitch βR or slow-tonic α’ or β’ fibers. The former corresponds to Type I fibers and the latter belongs to Type IIIA or IIIB fibers in mammals, respectively (Table 1) [3,7].

The ALD muscle (*M. anterior latissimus dorsi)* from the dorsal anterior position contains almost entirely slow-tonic fibers and responds to denervation with marked hypertrophy, but its tension developed in response to the application of K^+^-rich solutions is reduced by about 50% [9]. The sartorius, adductor and iliofibularis muscles from the leg are mixed-type composed of five types of fibers, αW, αR, βR and two types of slow-tonic fibers. In contract, posterior iliotibialis muscle contains predominantly fast-twitch fibers [10].

In the cross section of normal pectoralis muscles, many polygonal muscle fibers are bundled together and wrapped in a thin layer of connective tissue covering. Most of the muscle nuclei are located at the periphery of each fiber (Figure 1b). A marked pathological change in dystrophic pectoralis muscles are: a hypertrophy of muscle mass with fiber size variation, a proliferation of myofiber nuclei and satellite cells, cytoplasmic vacuolization, necrotic destruction of muscle fibers and the presence of ring fibers and fibrosis with fatty infiltration where muscle fibers are replaced by connective tissue [11,12,13,14,15] (Figure 1c).

The inheritance pattern was initially thought as an autosomal recessive mode designated as abnormal muscle (*am/am*) for homozygotes [1,11]. However, because dystrophic phenotypes are often seen in heterozygous muscles, they came to be identified as a co-dominant inheritance designated a gene symbol *AM/AM* [16,17]. Chicken muscular dystrophy is transmitted by a single gene, but the phenotype is modified by other background genes [16,17,18,19]. One of several lines of dystrophic New Hampshire chickens, line 413, was introduced with normal line 412 from the University of California at Davis to Japan in 1976 [17].

A traditional approach to the gene function sets about a phenotype analysis approach to a gene that encodes the phenotype. Before 1980, until advances in DNA technology based on the positional cloning and reverse genetics, very few human genes had been identified as disease loci. Despite the accumulation of experimental data obtained by these studies, the etiology of Duchenne muscular dystrophy has been unidentified for many years.

The reverse genetic approach clarifies abnormal cellular functions and various disease phenotypes from gene mutation. For example, the genetic analysis and positional cloning revealed a dystrophin gene mutation responsible for the Duchenne muscular dystrophy in 1987 [20]. The positional cloning for the genetic screenings opened new avenues to identify the gene mutation responsible for the chicken muscular dystrophy. Matsumoto et al. (2008) identified a *WWP1* gene mutation that led to an arg441-to-glu (R441Q) substitution in chickens with inherited muscular dystrophy [21]. The WW domain containing E3 ubiquitin protein ligase 1 (WWP1) is one of the ubiquitin ligases which play an important role in ubiquitin-proteasome pathway. This review discusses how mutated WWP1 protein functions in muscle fibers and leads to the above abnormal phenotypes.

## 2. Muscle Fiber Formation

The fiber types in chicken with muscular dystrophy are changed in a characteristic manner. Pathological lesions within fibers occur selectively in the fast-twitch αW fibers, but the phenotypic effect can be modified by the presence of fast-twitch αR and slow-twitch βR fibers neighboring them [3]. In this point of view, the following questions are given; (1) How are muscle fiber types specified during embryonic myogenesis? (2) What neuronal factors are involved in the changes of fiber type composition? (3) Why are fast-twitch αW fibers especially vulnerable for the pathological conditions under this myopathy?

**Figure 2:**
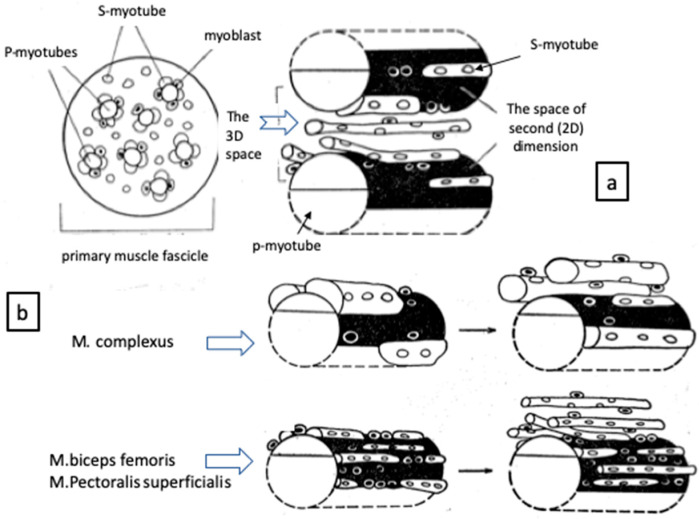
Schematic model indicating the stereographic structure of the relationship among primary myotube (p-myotube), secondary myotube (s-myotube) and myoblasts proliferating within a primary muscle fascicle (**a**). The s-myotubes take gradually the 2D space on the surface of p-myotubes. They separate from p-myotubes and then occupy the 3D space within a primary muscle fascicle (a) (right). (**b**) A comparison of myotube formation between complexus and other muscles. Note that a remarkable growth of s-myotubes in complexus muscle loses both 2D and 3D spaces rapidly to develop around p-myotubes compared to other muscles [22,24].

The mononucleated cells which engage in myosin synthesis are the myoblasts proper. The myoblasts continue to elongate and then begin to fuse with each other or with immature multinucleated myotubes to form syncytial myotubes to develop primary myotubes (p-myotube) which attach to muscle tendon at both ends. The surface of p-myotubes are used as a scaffold for the formation of secondary myotubes (s-myotube), which separate successively from p-myotube with the appearance of basal lamina between two myotubes. New myotubes are made from the surface of p-myotubes in sequence if the space is enough for the surface and periphery of p-myotube (Figure 2a) [22,23,24,25,26].

To prove such myogenic events, the complexus muscle which originates from dorsal spine of cervical vertebrae 3–5 and inserts on the posterior edge of the parietal bones [27] was compared with pectoralis and biceps femoris (*M. biceps femoris)*. The complexus muscles hypertrophy as it gets closer to the hatching time, followed by a rapid atrophy soon after hatching and become a mixed-twitch muscle [7,22,24]. This hypertrophy and atrophy axis shown at hatching is considered to be necessary for breaking the eggshell to hatch and thereafter for raising and lowering of the head to peck bait [27]. As compared the complexus muscle with others, the growth of s-myotubes separating from p-myotube was already obvious even in earlier stages of development. The primordia of primary muscle fascicles are clearly identified in embryonic muscles at 10 days in ovo (Figure 2b). As mentioned above, various myotubes are observed in a given primary muscle fascicle; p-myotubes locate inside and provide the 2D space on their surface as a place for growing s-myotubes and increasing myoblasts. The p-myotubes tend to be larger in size than s-myotubes around, but they are nearly equal between muscles [22]. In contrast, the s-myotubes growing on the surface and separating from p-myotubes gradually occupy both 2D and 3D spaces within a given primary fascicle. The s-myotubes do not fuse laterally with p-myotubes [23]. The results suggest that s-myotubes in the complexus muscles are larger in size but smaller in number compared to other muscles. Such reciprocal relationship between size and number of myotubes can be seen as early as 12–14 days in ovo which were also adapted for the total number of muscle fibers contained in a given muscle mass [24].

Ashmore et al. (1972) examined prenatal development of myotubes in fetal lambs and found two types of myotubes: the large p-myotubes were destined to be slow-twitch βR fibers, whereas s-myotubes tended to differentiate into fast-twitch αW or αR fibers [28]. During the myogenesis, the pattern of fiber types changed as a result of either a selective loss of β fibers or a rearrangement of some of the initial neuromuscular contact [29,30]. In support of our findings, McLenan (1983) suggested that each βR fiber serves as a structural framework around which small αW fibers develop [31]. In chicken pectoralis muscles which are devoid of βR fibers but contain α fibers with a substantial proportion, the αR fibers were thought to be raised from p-myotubes [7]. Although αW fibers in dystrophic pectoral muscles are earliest and severely affected, the transformation of aerobic αR fibers to αW fibers remain incomplete and tend to increase in their proportion with days after hatching. The αR fibers in dystrophic fast-twitch muscles indicate hypertrophy and contain more than normal concentration of nuclei, mitochondria and sarcoplasmic reticulum [3,7].

Most leg and neck muscles have mixed-fiber types containing oxidative aerobic fibers and exhibit little or no transformation to αW fibers, resulting in a minimal clinical involvement from this myopathy [7]. The effect of fiber types on dystrophic response suggests pathological lesions can be greatly modified by the type of neighboring fibers [3]. In this contention, the oxygen delivery from capillary network to muscle fibers was thought as an idea to improve myopathic characteristics. A modified azo dye coupling technique was used at pH 9.5 [32]. Localized areas of high enzymatic activity were found in specific and well-defined areas along the terminal arterial tree and capillary endothelium [33]. The therapy to newly hatched dystrophic chickens for six weeks resulted to retard effectively the early microvascular lesion and pathologic progress in dystrophic pectoralis muscle [33,34]. The authors noted that oxidative slow fibers are surrounded by rich capillaries with extracellular components which will help nearby fast-twitch fibers by supplying nutrition and oxygen, as discussed above.

## 3. Are Dystrophic αR Fibers Akin to Embryonic or Denervated?

There is evidence that dystrophic and denervated fibers of chicken pectoralis muscles recapture the nature of embryonic fiber type throughout the life in the isoform patterns of several myofibrillar proteins. Since around 1980, the method of two-dimensional gel electrophoresis in combination of immunoblotting was used frequently for the analysis of myofibrillar proteins in the developmental and diseased muscles. It was generally accepted that, when neonatal muscles are denervated, the protein isoform transition from neonatal to adult state is interrupted, whereas the denervation of mature muscles caused the reappearance of the neonatal forms of proteins. Specifically, some references are listed as follow: tropomyosin [35,36], troponin T [36], myosin heavy chain [37,38] and C-protein [39,40].

Briefly, normal embryonic pectoral muscles contain tropomyosin of both α- and β-chains followed by the expression of only α-chain ex ovo. However, β-chain of tropomyosin in dystrophic pectoral muscles reappeared 27 days after hatching and continued thereafter to be with α-chain in growing muscles [35]. Likewise, the slow and fast C-proteins are expressed in normal pectoralis muscles at late embryonic and neonatal period, but the slow C-protein disappeared ex ovo, leaving continued expression of only fast C-protein [40]. In the dystrophic pectoralis muscles, however, muscle fibers expressing slow C-protein reappeared about one-month ex ovo and increased with the progression of muscular dystrophy [39]. The adult dystrophic pectoralis muscles continue to express neonatal myosin heavy chain long after it disappeared from adult normal muscles. These results indicate that chicken muscular dystrophy inhibits the myofibrillar protein genes switching that normally occur during muscle maturation. Such phenomena resemble denervated or regenerating immature muscles which express embryonic myofibrillar proteins [41,42].

Acetylcholinesterase (AChE) localizes focally at the motor endplate of fast twitch fibers in normal biceps muscles, while AChE activity distributes symmetrically outside the motor endplates in dystrophic fibers. Sarcoplasmic AChE is maximal around the sites of motor endplates and declines in a regular fashion to either side. It is, however, present throughout the length of fibers in dystrophic biceps muscles [43,44,45].

**Figure 3:**
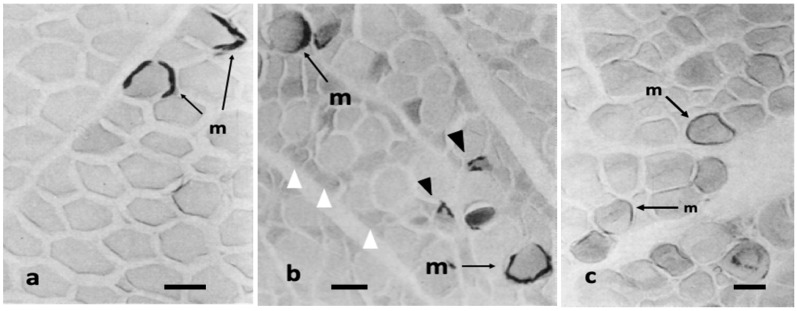
Transverse sections stained for the acetylcholinesterase (AChE) activity with histochemical method at motor endplates (m) of: normal (**a**); heterozygous (**b**); and dystrophic (**c**) pectoralis muscles. The AChE activity in normal pectoralis muscle fibers is confined to the motor endplates, while it extends to the extrajunctional sarcoplasm diffusely in heterozygous and dystrophic fibers. The hypertrophied αR fibers are surrounded by atrophied αW fibers (white arrowheads in (**b**)), some of which have AChE positive endplates (arrowheads in (**b**)). The majority of dystrophic fibers (**c**) are hypertrophy and contain intense AChE activity in sarcoplasm and have motor endplates (m), which are stained weaker and thinner than those in other genotypes. Note a positively stained sarcoplasm, likely “Ring fiber”, at the right lower corner (**c**), Bars = 50 μm. [17].

The AChE activity in normal pectoralis muscle is the same as in normal biceps muscle mentioned above (Figure 3a). The heterozygous pectoralis muscles are of interest in that: (1) hypertrophied αR fibers have a high AChE activity in motor endplates and sarcoplasm, but not extend to the extrajunctional regions as in normal motor endplates; and (2) the growth of αW fibers around αR fibers is strikingly retarded with intensive AChE activity diffused throughout the length of fibers (Figure 3b). These findings are in contrast to dystrophic pectoralis muscles which have round fibers, and it is hard to identify αR fibers only by AChE activity, which is highest in the motor endplates and extends to over a major portion of sarcoplasm. As mentioned by Ashmore et al. (1978), most end plates in dystrophic fibers are thin and partially fragmented [6]. Affected fibers vary in size and there are wider gaps among themselves (Figure 3c). Unlike αW fibers in dystrophic muscles, those in heterozygous muscles are capable of maintaining relatively normal aerobic enzyme activity regardless of abnormally high AChE activity.

From the results in heterozygous dystrophic muscles, the abnormal AChE reaction in αW fibers precede to the changes in mitochondrial enzyme reaction and cellular hypertrophy seen in homozygous dystrophic αW fibers. Moreover, αR fibers in heterozygous pectoralis muscles associate with a marked elevation of oxidative enzyme activity and hypertrophy response, suggesting that αR fibers are in the state of compensation for αW fibers weakened by this time [16]. An additional possibility considered is that αR fibers in heterozygous pectoralis muscles are inherently adapted much more efficiently to aerobic energy production than those in normal muscles, leading to increase in the number of mitochondria to make powerful aerobic energy in substitution for αW fibers [46,47]. Although αW fibers intend to produce glycolytic energy necessary for the movement after hatching, they become too immature to produce enough energy in cooperation with neighboring αR fibers. The αR fibers produce instead huge aerobic oxidative energy as a result of supplementation of αW fibers. As the disease progresses, some of αW fibers can revive from a war of attrition, while others become worse. The synthesis of many muscle proteins is greatly accelerated, leading to disability to cope with this failure of regulation and degenerative events ensue. The αR fibers are worn out and disintegrate as the energy burns out [6].

Similar to normal fast twitch fibers, most fibers in dystrophic biceps muscles result in marked atrophy and disappearance of hypertrophied fibers by 25 days after denervation [44]. However, a considerable number of hypertrophied fibers are found in adult pectoralis muscles at 25 days after denervation. Most hypertrophied fibers are identified as an aerobic αR fiber type on the basis of histochemical examination [48]. These results lead to the consideration that αR fibers in dystrophic pectoralis muscle might be less dependent on neural influences and resemble a long-lasting hypertrophy after denervation in slow tonic fibers in ALD muscle or the βR fibers in the mixed-type muscles [49,50]. It has also been demonstrated that various properties of dystrophic pectoralis muscle resemble those of slow-twitch muscles [51] and of immature or embryonic muscles in several myofibrillar proteins [36]. Considering these reports, most hypertrophied αR fibers in denervated dystrophic pectoralis muscle are more akin to be slow fibers [48].

**Figure 4:**
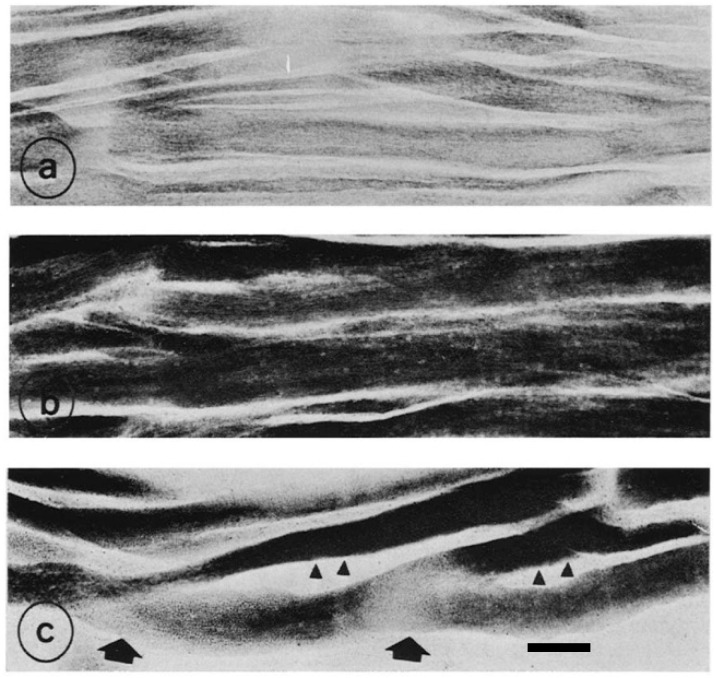
Longitudinal section of succinic dehydrogenase (SDH) activity in: normal (**a**); dystrophic (**b**); and combined (normal + dystrophic) donor muscles (**c**), regenerating in normal host chicks at 56 days post-operation made at 10 days after hatching. The SDH activity in dystrophic fibers is higher than in normal fibers. Compared with homogeneous enzyme reaction in normal and dystrophic donor muscles, SDH activity in combined transplants is more variable along the length, higher (arrowhead) and lower in others (large arrow). Adopted from Kikuchi et al. 1980 [47]. (**c**) Bar = 100 μm.

To confirm whether chicken muscular dystrophy is caused by myogenic or neurogenic factors, the hybrid multinucleated muscle fibers, derived from both normal and dystrophic donor muscle fragments, were produced [47]. The inherently different myoblasts responded differentially for the mitochondrial succinic dehydrogenase (SDH) activity and growth rate under the neural influences transmitted from host nerve (Figure 4). Ashmore and Doerr (1970) extracted mitochondria from both genotypes and found that dystrophic pectoralis muscle has a significantly higher concentration of mitochondria with a higher basal activity stabilizing to a greater degree than normal pectoralis muscle [46]. Considering that mitochondria are semiautonomous organelles under nuclear and cytoplasmic genetic control [52], it is of interest to address how such pre-existing mitochondria increase so greatly in number in dystrophic muscle fibers. The combined transplantations include mitochondria derived from the dissimilar genotypic nuclei. As myogenesis proceeds, the hybrid myofibers respond differentially for either sites with regional differences in mitochondrial SDH activity along their length (Figure 4c). The characteristics of high SDH activity with fiber hypertrophy might be due to an expression of dystrophic nuclei. The combined transplantation can regenerate mosaic myofibers in SDH reaction, confirming the previous report that this myopathy is not due to neurogenic [53].

## 4. Genetic Linkage Analysis of *AM* Gene

To narrow down the *AM* gene (capitalized due to dominant inheritance) candidate region by positional cloning, a resource family was produced from a subset of 55 backcross chicks from one F1 female. The pectoralis muscles were removed to detect *AM* phenotypes, stained with hematoxylin and eosin and serial sectioned with Van Gieson’s Sirius red F3BA (see Figure 1b,c) to specify pathological lesions. The amplified fragment length polymorphism (AFLP) locates *AM* gene to chicken chromosome 2q using a linkage map constructed with the Kobe University resource family [54]. The region including *AM* locus shows synteny with human chromosome 8q11-24.3 [55]. The *AM* gene is mapped 130 cm from distal end and narrowed down to approximately 4.0 Mbp including 34 functional genes [56,57].

In the second step, the haplotype analysis was carried out for 240 F2 individuals. The *AM* candidate region is now approximately 1.8 Mbp including 21 functional genes. The genotypes of 487 F2 individuals were analyzed using 11 additional markers to reduce the candidate region to within approximately 1 Mbp, which contain seven functional genes predicted as the most likely candidates: LOC420214, LOC428367, LOC420211, LOC420213, MMP16, ATP6VD2 and WWP1 in chicken chromosome 2q [58]. The sequence comparison and analysis of seven genes in normal and dystrophic chickens revealed that the *WWP1* missense mutation was predicted to influence the function of the WWP1 protein. The *WWP1* missense mutation was not found in 111 normal birds from 16 strains and highly conserved among tetrapods [21,59].

The WWP1 regulates protein trafficking and degradation, cell proliferation, apoptosis, signaling, transcription and viral budding [60]. Moreover, it is implicated in several diseases, such as cancers, infectious diseases, neurological diseases and aging. Northern blot analysis shows a high expression of transcripts in skeletal and cardiac muscles [61]. Full-length of chicken WWP1 shares 83% amino acid identity with human WWP1 protein [62]. The R441Q substitution occurred between WW domains 1 and 2 within a region highly conserved among tetrapods and snakes, including 100% conservation of 20 amino acids immediately surrounding R441. The WWP1 mutated in the coding region of the protein provides the most likely candidate responsible for causing chicken muscular dystrophy.

## 5. WWP1: E3 Ubiquitin Ligases

**Figure 5:**
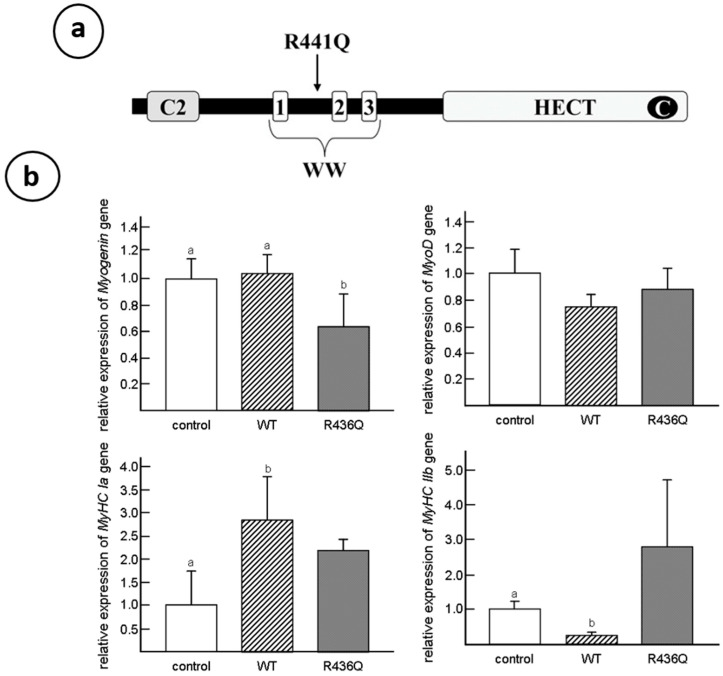
(**a**) The schematic domain structure of chicken WWP1 and the site of missense mutation. Chicken WWP1 protein is composed of 922 amino acids indicating WWP1 functional domains: C2 domain, three WW domains and HECT domain. C in HECT domain indicates an active cysteine residue. The arrow indicates the site of missense mutation. WW domains bind proline-rich region. (**b**) Muscle-differentiation markers (*Myog*, *MyoD*, *MyHC Ia* and *MyHC IIb*) in *WWP1*-transfected (WT and R436Q) and empty vector-transfected (control) C_2_C_12_ cells. Note that the R436Q-transfected cells retained the high expression of both slow *MyHC Ia* and fast *MyHC IIb* isoforms compared to control cells. *Y*-axis indicates relative expression level of each gene to the GAPDH gene expression. Different letters indicate significantly differences (*p* < 0.05) among column graphs. Adopted from Matsumoto et al. 2008 and 2010 [63].

The WWP1 protein is one of the HECT (homologous to E6AP carboxyl terminus)-type E3 ligases which play a key role in the ubiquitination cascade, and it is also responsible for the substrate recognition and modification with specific polyubiquitin chains. Protein ubiquitination is driven by the ubiquitin–proteasome system, involving three main groups of enzymes: E1 (ubiquitin-activating enzyme), E2 (ubiquitin-conjugating enzyme) and E3 (ubiquitin-protein ligase). This group of E3 ubiquitin ligases is composed of nine members including WW domain-containing E3 ubiquitin protein ligase 1 (WWP1) and the same class of ligase 2 (WWP2) [60]. The WWP1 is the most prominent among the family members tested. They are characterized by a similar domain structure with an N-terminal, membrane interacting C2 domain, 2–4 WW domains (three domains in the case of chickens) and a C-terminal HECT domain [61] (see Figure 5a). Despite intensive studies on oncogenic characters, the role of WWP1 to muscular functions has not yet been fully understood.

As mentioned above, the myofibrillar isozymes such as tropomyosin, troponin- T, myosin heavy chain and c-protein of dystrophic fast twitch muscles are unable to switch from embryonic to adult type. To confirm the effect of R430Q-mutated *WWP1* gene on myosin heavy chain (MyHC) gene expression, the normal or mutated *WWP1* gene were transfected into C_2_C_12_ mouse myoblasts to analyze the levels of muscle differentiation markers by real-time PCR [63]. The MyHCs are muscle proteins increasing during the myogenesis [64], which are divided into two classes: the Type I and Type II fast twitch fibers [3]. When excessive normal *WWP1* gene is transfected into C2C12 cells, the expression of slow myosin heavy chain isoform (MyHC Ia) gene is enhanced while fast IIb isoform (MyHC IIb) gene is only trace expressed. However, the R430Q-mutated *WWP1* gene transfected into cells expresses high levels of both slow-MyHC Ia and fast-MyHC IIb isoforms in C_2_C_12_ cells (Figure 5b). These results indicate that normal *WWP1* gene promotes switching predominantly to fast twitch isozyme expression whereas R430Q-mutated *WWP1* gene results to express both slow and fast isoforms, which is a characteristic of dystrophic pectoralis muscles [63]. The dystrophic phenotypes of chicken muscular dystrophy should be triggered by aberrant regulation of some WWP1 substrates, which was unknown until Cho et al. (2018) identified β-dystroglycan as a substrate of WWP1 [65]. They found that a missense mutation in *WWP1* gene is more effective at decreasing β-dystroglycan than normal WWP1 and reduces mutated WWP1 via the overactivation of autoubiquitination.

The antibody specific chicken WWP1 protein indicates that WWP1 in normal muscle is detected as ~130-kDa protein localizes to various structures, such as most conspicuously around sarcolemma, as well as at sarcoplasmic reticulum, mitochondria and nucleus [66]. The thickness of sarcolemma signals against to WWP1 antibody is bigger in normal pectoralis fibers than that in dystrophic fibers which contain mitochondrial signals distributed much more densely compared to those of normal pectoralis muscles. The degradation of aberrant WWP1 in sarcolemma is already obvious in dystrophic muscles seven days after hatching, and ~50% of full-length WWP1 is degraded during the prepathological stages, indicating that the R441Q missense mutation in WWP1 protein may play a role in the pathogenesis of chicken muscular dystrophy. Contrary to normal pectoralis muscle fibers, the sarcolemma of slow-tonic ALD fibers show weak WWP1 signals, while sarcoplasmic structures and nuclei are clearly labeled with WWP1 antibody. Accordingly, the differences of WWP1 signal intensity between normal and dystrophic ALD fibers are not so clear. The analysis also indicated that WWP1 protein expression in normal ALD fibers is much lower than that of normal pectoralis fibers.

As mentioned in the neuropathological section reported by Wilson et al. (1968), the dystrophic muscle fibers have an intense AChE activity present within sarcoplasm over a considerable extent from their motor endplates, as well as at the endplates, while normal fibers exhibit a high level of AChE restricted to the region of motor endplates [17,43,44,45,53]. Although atrophy is a typical response of normal fast twitch fibers upon denervation, a long-lasting post denervation hypertrophy in the slow muscle fibers in ALD and other muscles of mixed type was reported [9,49]. It has been demonstrated that many properties of dystrophic muscle resembled those of slow muscle [51] and of immature or embryonic muscle [44]. Moreover, Fambrough and Devreotes (1978) [67] and Ashmore et al. (1978) [6] suggested that acetylcholine receptor (AChR) molecules are transported from the interior of cell to sarcolemma, and both AChE and AChR molecules are transported together in granular form to any surface available in the cell.

During muscle development, the agrin concentrates along the synaptic basement membrane where it can interact with the postsynaptic membrane to form AChRs at sites of nerve–muscle contact [68,69]. The AChRs aggregate to mediate the formation of AChR clusters which contain a member of the dystrophin-associated protein complex including a core molecule, β-dystroglycan. During early embryogenesis, AChRs are widespread over the entire sarcolemma, but are gradually concentrated in clusters at motor end plates [70,71]. The β-dystroglycan is necessary for the condensation of AChR microclusters and stabilization of AChRs within the plasma membrane, as well as for the assembly of AChE. These results highlight the importance of β-dystroglycan in synapse formation.

As muscle development proceed, AChRs begin to migrate laterally from extrajunctional membrane regions and also insert into the membrane of newly synthesized AChR molecules (for reviews, see those of Fambrough [72] and Schuetze and Role [73]). The myotubes differentiated from β-dystroglycan null embryonic stem cells cannot form AChR clusters, which are 2–3 times wider, about half as dense and less stable than those on β-dystroglycan +/+ myotubes [74]. The AChRs at motor endplates are similarly affected and increased in nerve terminal size with less AChE at these junctions. Taken together, the denervation of immature fibers seen in dystrophic muscles can be explained as β-dystroglycan is perturbed by excess-ubiquitination of aberrant WWP1 protein with R441Q missense mutation and cannot perform normal maturation and stabilization of AChRs and AChE at motor endplates.

## 6. Caveolin-3: Another Causative Process of Muscular Dystrophy

Caveolae are flask-shaped vesicular invaginations of plasma membrane which are known to regulate endocytosis, exocytosis, cholesterol homeostasis, signal transduction and mechanoprotection [75]. Caveolin-3 (Cav-3) is the structural protein component of caveolae in skeletal and cardiac muscle cells [76,77] and promotes proper clustering of AChRs but diffuses the distribution of AChRs in myotubes deficient Cav-3 [77,78]. Although Cav-3 is predominantly associated with sarcolemma of mature muscles, it distributes with the transverse tubule (T-tubule) system in differentiating myotubes [79,80]. The T-tubules of Cav-3 null mice are dilated and run in irregular directions, suggesting that Cav-3 is involved in the organization of T-tubules but not essential for their formation [81]. Cav-3 deficiency induces a muscular dystrophic phenotype (Rippling disease) [82], while its overexpression does harm to muscle tissue [83,84,85]. Cav-3 overexpressing transgenic mice revealed severely affected symptoms with an increase in the number of sarcolemmal caveolae, which is one of the Duchenne muscular dystrophy traits in humans, and the downregulation of dystrophin and β-dystroglycan protein expression [84].

**Figure 6:**
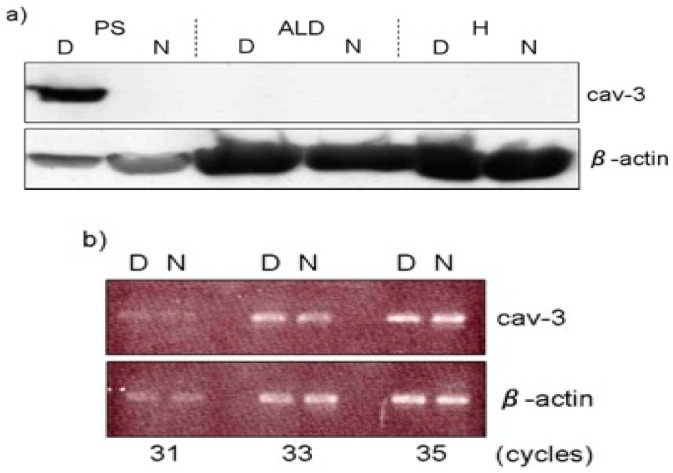
Expression of caveolin-3 (Cav-3) at protein and mRNA level. (**a**) Expression of Cav-3 in M. pectoralis superficialis (PS), M. anterior latissimus dorsi (ALD) and heart (H) was analyzed by Western blotting. Note that PS expressed higher amount of Cav-3 protein (7.12 ± 3.31-fold) in dystrophic chickens (D), while the expression in ALD and H was undetectable as in normal chickens (N). (**b**) The semi-quantitative RT-PCR analysis indicated that its mRNA expression was at the similar level between dystrophic (D) and normal (N) pectoralis muscle. Adopted from Matsumoto et al. 2010 [85].

Moreover, these mice show elevated blood serum creatine kinase (CK) levels and are consistent with the marked elevation of this enzyme in chicken muscular dystrophy [86]. In addition, serum pyruvate kinase (PK) activities in dystrophic chickens were approximately 30-fold higher than those in normal chickens, while a seven-fold elevation was detected in serum CK activity at 37 days of age (Table 2) [17]. It is thought that the rise of serum CK and PK causes lysis or necrosis of muscle fibers, with subsequent release of these enzymes into the blood [16]. The caveolae function in buffering and resealing mechanical stresses at the plasma membrane, including sarcolemma and T-tubules system [87]. The freeze-fracture morphology of sarcolemma in soleus muscle in Cav-3 −/−, Cav-3 +/− and Cav-3 +/+ wild-type mice revealed that caveolae were abundant in wild-type mice, less frequent in heterozygous Cav-3 +/− mice and scarce in Cav-3 −/− mice [82]. The pectoralis muscles were investigated in normal and dystrophic chickens at embryo, early post-hatching and adult stages [88]. The average densities of caveolae are higher (30 /μm^2^) in adult dystrophic fibers than those (17 /μm^2^) in age-matched normal fibers. The distribution pattern of caveolae in dystrophic fibers is random arrangement compared to rectangular one in normal fibers. These abnormalities were already obvious at seven days after hatching before the appearance of clinical symptoms. In contrast, the sarcolemma of slow tonic ALD fibers have randomly dispersed caveolae whose appearance and distribution are unaffected throughout the life by this myopathy [89]. Matsumoto et al. (2010) reported the expression of Cav-3 and other caveolae-related proteins in adult normal and dystrophic chickens (Figure 6a). Western blotting and semi-quantitative RT-PCR analysis revealed that Cav-3 is higher only in affected fast-twitch muscles of dystrophic chickens and the amount of caveolin-3 protein is regulated in posttranslational modification, since no significant increase is observed at the mRNA level (Figure 6b).

Cav-3 and dystrophin bind to the same site of β-dystroglycan and interact competitively with β-dystroglycan [90]. It was reported that WWP1, which is a primary causative protein of chicken muscular dystrophy, also requires its site in β-dystroglycan. Cav-3 and dystrophin compete against WWP1 as well to negatively affect WWP1-mediated β-dystroglycan degradation [65]. Accordingly, these results explain why the overexpression of Cav-3 induces the destabilization and degradation of the DGC complex, leading to major defects in membrane integrity and intracellular myofibril alignments. 

As mentioned above, caveolae play a role in buffering mechanical stress at the plasma membrane, since muscle fibers are repeatedly experiencing mechanical stress at the plasma membrane, which must be able to rapidly repair wounds. The failure to repair causes the degenerative changes in Z-band and myofibrils in embryonic pectoralis muscle fibers [91]. McLean et al. (1986) also found extensive changes in patterning of sarcolemmal caveolae of chicken dystrophic PLD muscle, but the patterning of normal fibers is arranged in striking bands over the myofibrillar I-bands [89]. These morphological changes in caveolae structure and distribution are already detected in dystrophic pectoralis fibers as early as seven days after hatching [88]. Together, results indicate that the overexpression of Cav-3 protein is involved in another causative process of chicken muscular dystrophy.

The primary cause of chicken muscular dystrophy is due to an aberrant WWP1 protein which targets β-dystroglycan as substrate. The β-dystroglycan is a core molecule of the DGC and guarantees stability to sarcolemma [92]. Loss of β-dystroglycan in dystrophic fast-twitch fibers are vulnerable to contraction-induced wounding and are likely to undergo repeated cycles of injury and repair. Caveolae provide reserve force of expandable membrane when tension is added. Stretching of muscle caused a loss of caveolae, apparently by their flattening [93], and a similar effect, “unfolding” of caveolae, was proposed in endothelial cells upon changes in capillary volume [94]. Caveolae were shown to flatten in response to changes in membrane tension, both upon cell swelling or with stretch. This process was energy-independent and caused release of cavins from the caveolae, raising the possibility that cavins may act as cytosolic signals for changes in membrane tension [95].

Feit et al. (1985 and 1989) measured both tension and stiffness as a function of muscle length under relaxing conditions on isolated small bundles of chemically skinned pectoralis myofibers from normal and dystrophic chickens aged between 45 and 55 days. They indicated that dystrophic pectoralis muscles show increased proportions of high-molecular-weight collagen, suggestive of increased cross-linking and are stiffer than normal muscles, and develop more tension for the same amount of stretch [96,97].

Fujii, Murota and Tanzer (1983) found an increase in amount of total collagen with an increased proportion of Type III collagen in muscle as early as 13 days following progressive deterioration to 19 days of embryos. This suggests the production of more immature collagen fibers compared to normal ones. The stiffness is mediated by such altered form of collagen which is collagenase-resistant by virtue of excessive crosslinking [98]. Moreover, an ultrastructural study of the tendon in embryonic gastrocnemius muscles showed significant alterations in developing myotendinous junction from dystrophic chickens as early as 13 days of embryos [99].

Then, it is of interest to elucidate what happen if dystrophic muscles with considerable amount of Type III collagen fibers in extracellular space and deteriorated myofibrils in immature myotubes, are added the tension to some extent for various periods? The stretch-induced growth in chicken wing patagialis muscles were conducted by Ashmore’s group during three years after 1980. The patagialis muscles, one of the fast twitch muscles, were extended at six weeks of age for 1–5 weeks. The passive stretch is a powerful inducer of muscle growth and the sift of fiber types from αW to αR fibers as the percent of αR fibers had increased from 11% in the control to 43% in the stretched muscles [100,101]. It was also confirmed in rat and rabbit that a chronic increase in tension as a result of stretch applied by the lengthening procedure is a potent stimulus for fast-to-slow myosin transformation and for muscle hypertrophy [102,103].

The patagialis muscles of normal and dystrophic chickens at seven days ex ovo were stretched for six weeks [104]. The stretched dystrophic muscles increased in weight, muscle mass cross-sectional area and fiber cross-sectional area, revealing a protective effect of stretch against the progressive pathology of muscular dystrophy. Stretched dystrophic muscles contained a higher rate of slow-twitch αR fibers than normal stretched muscles. The dystrophic fibers showed a more striking hypertrophy and have a larger number of mitochondria with intense SDH activity than those in age-matched normal muscles, so that they were a more uniform oxidative fiber profile compared with normal fibers stretched.

As mentioned earlier, the combined transplantation of both normal and dystrophic muscle fragments produces a single hybrid myofiber in which normal and dystrophic nuclei coexist. The regenerating fibers indicate regional differences in oxidative enzyme and growth rate along their length [47]. These results were supported by the histochemical reaction of SDH activity in longitudinal sections of combined transplantation (see Figure 4). Although it is not clear whether the fiber hypertrophy and mitochondrial SDH activity in cytoplasm are programmed genetically, or occur as a secondary compensatory response, it appears that either of these changes is characteristic of stretched and transplanted muscles. It has been suggested that mitochondria from dystrophic pectoralis muscles have not only significantly higher concentration but also higher basal activity stabilizing to greater degree than normal pectoralis muscle [46].

From these points of view, it was revealed that β-dystroglycan ubiquitinated by excessive afferent WWP1 causes an accumulation of caveolin, stiffness and tension due to extracellular space with immature collagens and distorted myofibrils in dystrophic muscles beginning from in ovo stages. In addition, Lee et al. (2013) reported in a C_2_C_12_ cells in vitro study that WWP1 protein interacts with AMP-activated protein kinase (AMPK) and downregulates its expression through ubiquitin ligase activity in skeletal muscle [105]. The AMPK is a sensor of cellular energy change, maintains the energy balance by decreasing the ATP-consuming processes and associates with increased mitochondrial enzyme content and mitochondrial biogenesis in rat skeletal muscle [106,107]. The immunoactivity of WWP1 antibody to sarcolemma in dystrophic pectoralis fibers is weaker than in control pectoralis fibers, whereas dystrophic fibers contain mitochondrial signals distributed much more densely compared to those of normal pectoralis muscles [66]. This result might be associated with increased mitochondria and their higher enzyme activity in dystrophic fibers compared to those in normal fibers. It is necessary to prove whether abnormally ubiquitinated AMPK and related proteins in dystrophic chickens would increase necessary protein levels via a fiber type shift, resulting in more slow, oxidative fibers that are much more resistant to contraction-induced damage.

In 2005, the hypoglycosylation and laminin-binding of defective α-dystroglycan were implicated to trigger the onset of chicken muscular dystrophy [108]. However, there were no proteins associated to glycosylation in the AM region on chicken chromosome 2q and aberrant glycosylation does not appear to be caused by its direct interaction with mutated WWP1 protein. The short sugar chain of α-dystroglycan in dystrophic muscles might be an early phase of the secondary result of the pathological changes following deregulation of β-dystroglycan on sarcolemma.

## Figures and Tables

**Table 1:** **Classification of different fiber types in chicken muscle.** The twitch muscle fibers with high myofibrillar adenosine triphosphatase (ATPase) after alkaline preincubation and low activity after acid preincubation are called α fibers. They are divided further into two subtypes, αW fibers with low oxidative activity and αR fibers with high oxidative activity (succinic dehydrogenase, SDH) or nicotinamide adenine dinucleotide tetrazolium reductase (NADH-TR). The fibers with the reverse ATPase and high oxidative activity are called βR fibers. The tonic muscle is also heterogeneous in fiber types with respect to ATPase activity, which do not reverse in myofibrillar ATPase pattern when exposed to acidic or alkaline preincubation. The β’ fibers with more intense reaction tend to locate centrally among groups of lighter-staining α’ fibers in a similar way in twitch muscles, which consist of βR fibers located centrally within groups of α fibers. The classification in mammals was made by Brook and Keizer [8]. The multiple innervation of βR fibers in twitch muscles was reported for the first time by Ashmore et al. (1978) [5,6].

Muscle Fiber Types	Twitch Fibers			Tonic Fibers	
Ashmore and Doerr (1971) [2]	αW	αR	βR	α’	β’
Brooke and Kaiser (1970) [8]	||B	||A	|	|||A	|||B
Histochemical criteria					
ATPase (pH 10)	●	●	○	●	●
ATPase (pH 4.1)	○	○	●	◎	●
SDH or NADH-TR	○	◎	●	◎	●
Phosphorylase	●	◎	○	◎	◎
Innervation pattern	Focal	Focal	Multiple	Multiple	Multiple

Modified Barnard’s classification [3]. Enzyme activity is expressed as ● high, ◎medium and ○ low.

**Table 2:** **Blood serum pyruvate kinase (PK) and creatine phosphokinase (CPK) activities in normal, dystrophic and heterozygous carrier chicks ^a^**.

Enzyme	Age (Days)	Normal ^b^ (mU/mL)	Dystrophy ^b^ (mU/mL)	Heterozygote ^b^ (mU/mL)
PK	37	401 ± 111(8)	12,430 ± 6,269(4) ^c^	630 ± 209(5)
	70–86	405 ± 73(4)	10,773 ± 6,800(7) ^c^	1,213 ± 552(10) ^d^
	475	229 ± 33(7)	8,940 ± 4,032(5) ^c^	516 ± 138(7) ^d^
CPK	37	141 ± 55(8)	1,071 ± 812(4) ^c^	180 ± 31(5)
	70–86	164 ± 41(4)	1,146 ± 599(7) ^c^	183 ± (10)
	475	30 ± 9(7)	986 ± 586(5) ^c^	46 ± 10(7) ^e^

a: Chicks tested were White Leghorn breed. b: Mean enzyme activity ± SD. Number of chicks tested in parentheses. Statistical analyses were performed using paired t-tests compared with normal chicks. c: *p* < 0.01, d: *p* < 0.005, e: *p* < 0.05.
